# Vascular aging, the vascular cytoskeleton and aortic stiffness

**DOI:** 10.37349/emed.2021.00041

**Published:** 2021-06-30

**Authors:** Lova Prasadareddy Kajuluri, Kuldeep Singh, Kathleen G Morgan

**Affiliations:** 1 Department of Health Sciences, Boston University, Boston, MA 02215, USA; 2 CSIR-Institute of Himalayan Bioresource Technology, Palampur, Himachal Pradesh 176061, India

**Keywords:** Vascular aging, cytoskeleton, focal adhesion, aortic stiffness

## Abstract

Vascular aging, aortic stiffness and hypertension are mechanistically interrelated. The perspective presented here will focus mainly on the molecular mechanisms of age-associated increases in the stiffness of the vascular smooth muscle cell (VSMC). This review will highlight the mechanisms by which the VSMC contributes to disorders of vascular aging. Distinct functional sub-components of the vascular cell and the molecular mechanisms of the protein-protein interactions, signaling mechanisms and intracellular trafficking processes in the setting of the aging aorta will be detailed.

## Introduction

### Definition and quantification of vascular aging

Vascular aging has been stated to be a key clinical factor in the determination of the health of the vascular system [1]. With age there is known to be a trend, dependent on the patient’s lifestyle, toward progressive remodeling and, especially, stiffening of the vasculature [2]. This is of note because vascular stiffening can be associated with, and is thought to be a cause of, hypertension, stroke, and vascular dementia [3]. The relative pace of vascular aging has been described by the concept of vascular age determination according to comparison with key clinical guidelines to indicate the relative slowing or acceleration of vascular function deterioration [1]. Specifically, high-resolution B-mode ultrasound has been used to measure carotid artery intima-media thickness (CIMT) [4] and vascular age has been defined by comparison to the age at which the composite CIMT measurements would represent the median value in the previously published Atherosclerosis Risk in Communities study [4–6]. Thus, clinically, vascular aging is quantifiable, but in order to design therapeutics to prevent or reverse vascular aging, the cellular and molecular basis must also be determined.

Hypertension and aortic stiffness during aging are interrelated. It has been reported for the Framingham Heart Study Offspring cohort that increased *in vivo* aortic stiffness [measured by carotid femoral pulse wave velocity (CFPWV)] is a strong predictor of the progression of hypertension [7] and has been suggested to be “an inevitable accompaniment of isolated hypertension” [8]. In the large arteries such as the aorta and carotid, an increase in arterial stiffness is known to parallel general vascular aging and to be a major predictor of cerebral bleeds and end organ damage to the high-flow organs, the brain, heart, and kidneys, in general [9]. Furthermore, the stiffness of the aorta is a quantifiable biomechanical property of aortic tissue and readily amenable to the development of prototype potential therapeutic agents. Thus, we will focus here specifically on aortic stiffness during aging.

### Components of vascular stiffening with age

Both cellular and acellular factors are known to be involved in aging-associated changes in stiffness of blood vessels. It is well established that aging leads to a stiffening, specifically, of the extracellular matrix (ECM) via collagen crosslinking and elastin degradation [10–13]. Much has been previously written on this subject and hence, will not be discussed in detail here.

More recently, it has been shown that the vascular smooth muscle cell (VSMC) [14–17] also undergoes aging-dependent changes that increase its stiffness and, as a result, the stiffness of the aortic wall. Within the VSMC, the activity of the contractile filaments as well as the molecular signaling pathways that regulate actin polymerization and focal adhesion (FA) signaling, are sources of increases in vascular stiffness with age [18]. It has only recently been recognized that regulation of the stiffness of the cytoskeleton of the VSMC can contribute up to 50% of total aortic stiffness even in young adult aortas in mouse models [16]. Similar quantitative biomechanical data are much needed from live human tissues. In addition to VSMC stiffness, increased VSMC adhesion to the ECM has been shown to contribute to increased aortic stiffness with aging [19]. Furthermore, both VSMC stiffness and VSMC adhesion, but not changes in ECM composition, have been shown to potentially contribute to increased aortic stiffness in hypertension, which further increases with hypertensive aging [20, 21].

Since the endothelium is only a monolayer of cells, at first glance it may seem unlikely that its structure would cause major changes in the overall stiffness of the wall; however, the endothelium releases vasodilators (nitric oxide (NO) [22–24], prostacyclin [25], endothelium-derived hyperpolarizing factors (EDHFs, 11, 12-epoxyeicosatrienoic acid [26, 27]) and vasoconstrictors (endothelin [28] and thromboxane A2 [29]) that regulate the activity of the contractile filaments in underlying VSMCs, generally by affecting the intracellular calcium concentrations [30]. In healthy young and adult individuals, a balance between vasodilators and vasoconstrictors allows the vessel to undergo changes in its diameter that modulate the incoming pulsatile blood flow, however, this function is largely lost with advancing age, resulting in stiffening of the vascular wall and hypertension [31, 32]. Age-induced loss of endothelial function, particularly loss of endothelium-dependent vasodilation occurs mainly because of reduced nitric oxide bioavailability triggered by an increased stiffness of the endothelial cell cortex [33, 34] and increased oxidative stress in the vasculature [35]. Increased stiffness of the endothelial cell cortex decreases the release of NO from the endothelium [36] and increased oxidative stress causes an increase in reactive oxygen species (ROS) molecules, such as superoxide radicals, that scavenge nitric oxide [37].

The outer adventitial layer of aorta confers structural integrity and contains a cellular repertoire of fibroblasts, macrophages, dendritic cells, mast cells and vascular progenitor cells etc. [38] that may also dynamically alter the total aortic stiffness. Though the collagen fibers produced by adventitial fibroblasts are clearly involved in regulating the stiffness [39], the contribution of cytoskeletal structures of adventitial cells to aortic stiffness is unknown.

### *In vitro* handling of experimental tissues

There exists a large literature on changes in aortic stiffness due to aging of the matrix layers of the aorta [40–42] and thus will not be covered in detail here. Much less is known about aging of the VSMCs since, experimentally, the majority of studies on matrix utilize tissues from slaughterhouses that are studied hours, if not days, after transport to the research lab. Under these conditions, the VSMCs are likely malfunctioning, or are simply dead. It may be reasonable to assume that the matrix is preserved in the native state. However, it would also be worthwhile to determine whether post-translational modifications, fibroblast function, etc., are preserved in the matrix with long-term storage after removal from the animal. Additionally, cultured, but growth-arrested, VSMCs are also often used for study of the native aortic VSMCs but, clearly, even though they are growth-arrested, the cells will differ in morphology, and relative abundance of isoforms of contractile proteins and contractile ability, compared to the native cells in the aorta of a living human. Thus, we will focus here on the reported properties and aging of freshly isolated or in situ VSMCs where possible.

## Subcellular structures responsible for regulated contractility and stiffness of the vascular cell

In both the human as well as mouse models of aging, increased aortic stiffness is associated with a damaging increase in the pulsatility of the blood sent from the heart to the high flow organs, especially the brain, kidney, and the heart [40, 43–45]. Hence, the subcellular sites of generation of these sources of increased stiffness are important to identify since they may be sites where modulation may be therapeutically useful.

### The contractile filaments

The attachment of the smooth muscle myosin heads to the actin filaments in the contractile filaments leads to the generation of contractile force, vascular tone, and, also for the duration of the attachment, it increases the stiffness of the cell [46, 47]. Drugs that regulate smooth muscle myosin activity will regulate both stiffness and steady state blood pressure; however, the contractile filaments may not be a good choice for the design of therapeutic targets to decrease aortic stiffness since vascular tone and contractility will be decreased in parallel with stiffness. Though there are no reports of altered myosin activity with aging, elevated levels of alpha smooth muscle actin have been shown to contribute to increased VSMC stiffness with ageing [14]. Additionally, age-dependent increases in actin cytoskeletal stiffness have been shown to be positively associated with pro-fibrotic transforming growth factor (TGF)-β expression and this is reinforced through mechanosensitive integrin receptors on the cell surface [48].

### The nonmuscle actin cytoskeleton

**Figure 1.**
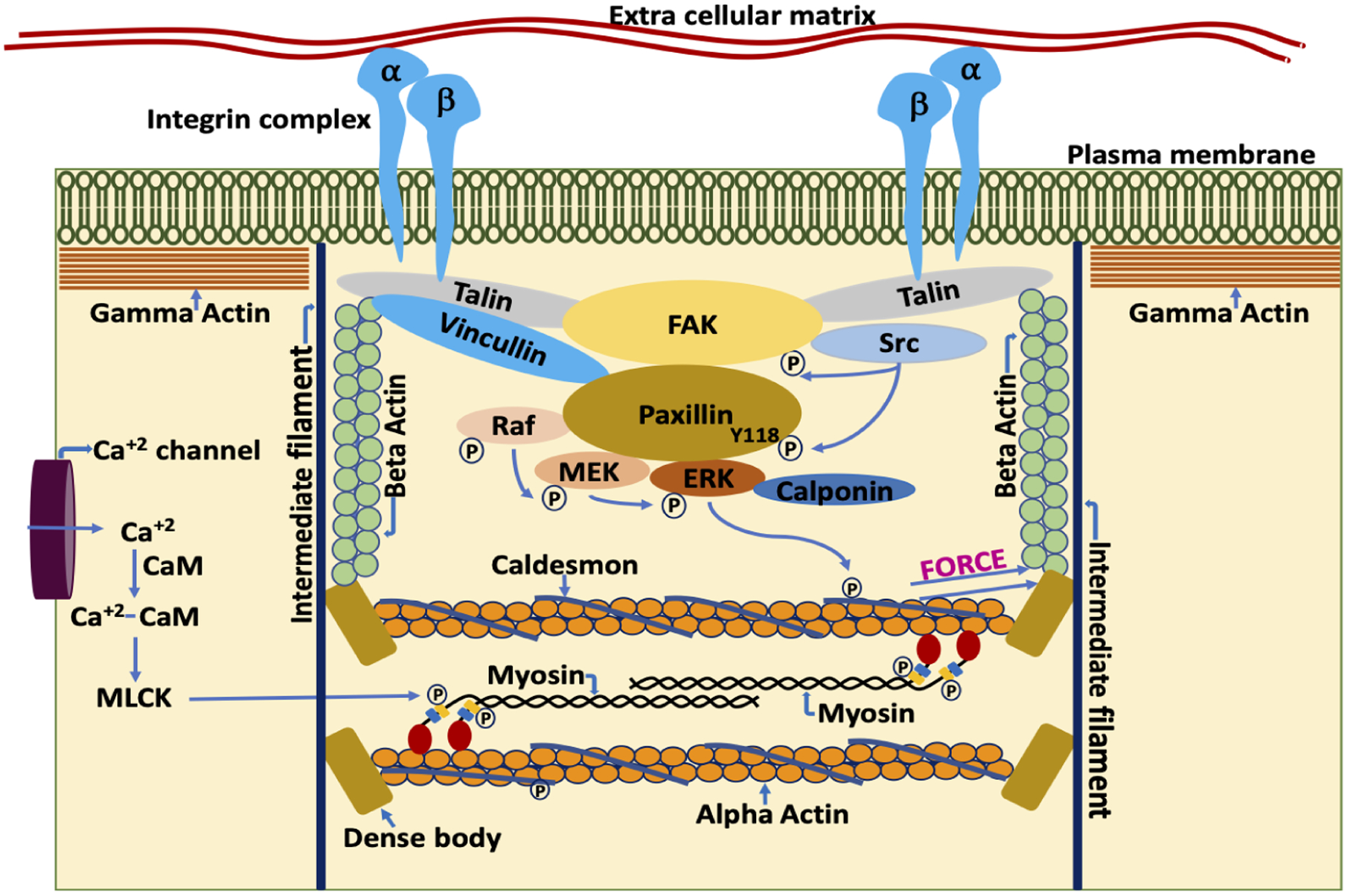
Schematic representation of components of the VSMC by which it regulates contractility and stiffness. The smooth muscle cell plasma membrane is spanned by FA complexes containing, among many other proteins, talin, vinculin, FA kinase (FAK), and integrins [52]. Cytoskeletal proteins of the FA complexes connect to the membrane-spanning integrins, composed of alpha and beta integrin heterodimers. On the cytoplasmic side, the FAs connect to the actin cytoskeletal filaments. Thus, the integrin complex connects the interior of the cell to the ECM allowing cell-matrix communication and signaling [53, 54]. Contractile force is generated by the actomyosin cross bridge cycle. During the cross-bridge cycle, force is generated by movement of myosin head domains while they are attached to the actin filaments. Acto-myosin cross bridge cycling is a tightly regulated process, involving both the thin and thick filaments. Thin filament regulation, in part, involves the blocking of myosin attachment sites on F-actin by caldesmon [55]. Caldesmon, in turn, is regulated by a complex, Src dependent signaling cascade. Src dependent phosphorylation of paxillin at Y118 allows the binding of rapidly accelerated fibrosarcoma (Raf) and extracellular signal-regulated kinase (ERK) to mitogen-activated protein kinase kinase (MEK) bound paxillin [56]. The formation of this complex leads to the activation of MEK by Raf and ERK transphosphorylation by active MEK. Subsequently, activated ERK translocates to, and phosphorylates, caldesmon [57]. Once phosphorylated, caldesmon undergoes a conformation change in its structure and no longer blocks the myosin attachment sites on F-actin. This sequence of events, then promotes acto-myosin interaction. However, attachment of the myosin head to F-actin is also regulated by phosphorylation of the myosin regulatory light chain (MLC), leading to additional signaling cascades described as thick filament regulation [58]. For example, increased intracellular calcium levels during agonist-induced opening of calcium channels in the plasmalemma leads to the formation of calcium-calmodulin complexes which then activates myosin light chain kinase (MLCK) [59–61]. Active MLCK then phosphorylates the myosin light chains which activates myosin ATPase activity [62]. Increased myosin ATPase activity leads to a conformational change in the head of myosin and promotes the attachment of myosin to actin in the strong binding conformation. Force generated during acto-myosin interaction is transmitted to dense bodies and through the nonmuscle actin cytoskeleton, to FA complexes, including the transmembrane integrins and, subsequently, to the ECM and the vessel wall [46, 63]. Simultaneous contraction of the VSMCs in the vessel wall leads to vascular constriction, which, when increased in extent or duration also leads to increased pathologies of hypertension and vascular stiffness [46]

VSMCs, unlike striated muscle cells, lack tendons, but transmit contractile force through the non-muscle cytoskeleton to FAs that span the plasmalemma and communicate force and stiffness to the ECM (Figure 1). This allows the matrix between cells to act as a sort of intramuscular tendon and to communicate contractile forces within the blood vessel or organ. VSMCs contain 3 isoforms of actin: alpha smooth muscle actin, beta nonmuscle actin and gamma nonmuscle actin [46, 49]. Alpha actin is located in the contractile filaments where it interacts with smooth muscle myosin cross-bridges during contractile activation. Beta actin is localized around the dense bodies, intracellular sites where contractile filaments terminate and gamma actin is present in the cell cortex [50, 51]. Force generated by the contractile filaments is transmitted from the dense bodies to a diffuse subplasmalemmal nonmuscle cortex containing nonmuscle gamma actin and the FAs. Both the beta actin cytoskeleton and cortical gamma actin cytoskeleton as well as the contractile filaments contribute to total smooth muscle stiffness.

### FA dynamics

FAs connect the vascular smooth muscle cytoskeleton to the ECM, but unlike the connection of striated muscle cells to tendons, vascular FAs are dynamic, multiprotein structures regulated by biomechanical forces as well as biochemical signaling [64, 65]. Furthermore, when mutations occur in the ECM protein, fibrillin-1, which normally links the vascular FAs and the extracellular vascular matrix, this can result in thoracic aortic aneurysms and dissections [66].

As indicated in Figure 1, the plasmalemma is spanned by integrin complexes connecting, extracellularly, with matrix molecules [53] and, intracellularly, with cytoskeletal complexes [54]. An important cluster (Figure 1) of FA molecules, including talin, directly contact the integrins [67] and, also link to FAK [68]. FAK, in turn links to Paxillin, and both FAK and Paxillin are phosphorylated by Src in a tension-dependent manner [56]. The posttranslational modification of these molecules and the interactions of these proteins has been observed to be quite dynamic in young mouse aortas as well as the smooth muscle of airways in young mice [56, 69–71]. Vasoconstrictors and other agonists can increase protein-protein interactions, and VSMC adhesion to ECM accompanied by cytoskeletal remodeling and hence, increase stiffness [17, 18, 72]. However, it has been observed in mouse models that the dynamic nature of these signaling mechanisms, which provides a sort of shock absorber for the cytoskeleton, is diminished by age [18] and that this contributes to the increased stiffness of aged aortas. Again, these concepts are, thus far, based on animal studies and similar studies using in human blood vessels are greatly needed.

Cytoskeletal elements also serve the function of regulating the formation of signaling complexes that, in turn, also regulate stiffness and contractility of the muscle cell. The Src dependent phosphorylation of Paxillin at Y118 is critical in regulating the scaffolding property of Paxillin, bringing together Raf, MEK and ERK [56] and leading to the phosphorylation of caldesmon [73] an actin binding protein that acts in a manner analogous to that of troponin in striated muscle and directly regulates the activation of the contractile filaments. ERK, when phosphorylated by MEK, translocates to the actin filaments of smooth muscle cells where it phosphorylates the inhibitory protein, caldesmon, causing a conformational change that disinhibits the contractile filaments [57]. This signaling pathway is often referred to as “thin filament regulation” in contrast to the parallel pathway of “thick filament regulation” by which a Ca dependent activation of MLCK leads to phosphorylation of the 20 kDa myosin light chains and activation of myosin motor activity. Both thin filament and thick filament regulation are needed for maximal contractile activation of the VSMC [46]. Importantly, both cross-bridge attachment in the contractile filaments (thick filament regulation) and the assembly of the ECM-integrin-cytoskeletal subplasmalemmal protein complexes (FA dynamics) have been shown to regulate the stiffness of the VSMC [16, 74–76], particularly in the proximal aorta.

### Cadherins

Unlike integrins which mediate cell-matrix attachment, cadherins mediates cell-cell attachment. Cadherins are calcium binding transmembrane proteins that connect internally to the actin cytoskeleton through adherin junctions composed mainly of catenins [77]. N-cadherin is the predominant cadherin that is expressed in VSMCs whose density and clustering was shown to increase with agonist treatment [78]. Both integrins and cadherins have been shown to engage in crosstalk mediating the mechanosignaling and determining the localization of cellular forces; however, most of these studies were performed on cell types other than VSMCs [79]. Future studies are required to understand how Integrins and cadherins crosstalk in regulating the contractile force transmission between cell-cell and cell-ECM junctions and how this would regulate cell stiffness.

### Other cytoskeletal elements

Other structural components of the contractile smooth muscle, such as intermediate filaments, to the best of our knowledge, have not been studied in the context of vascular stiffness and aging and, microtubules are scarce in contractile VSMCs and seem to have little acute function [80, 81].

## Epigenetic changes

Epigenetic alterations with aging have emerged as one of the crucial events that cause cardiovascular pathologies which include, among many, the stiffening of the aorta. Epigenetic changes modulate the expression of genes. These include DNA methylation, histone acetylation and chromatin remodeling. Long non-coding RNAs and short non-coding RNAs [microRNAs (miRs)] also act as epigenetic effectors [82]. In smooth muscle cells, methylation status of genes is regulated by DNA methyl transferases (DNMT3A, DNMT3B, DNMT1) and Ten-eleven translocation (TET) proteins. TET2 was shown to be a master epigenetic regulator of smooth muscle cell phenotypic modulation [83]. However, the role of TETs in cardiovascular aging remains elusive. A previous report from our lab showed DNA hypomethylation of miR-203 increased stiffness of the aorta with aging. This epigenetic change increased the expression of miR-203, which down regulates Src, a key tyrosine kinase required for FA signaling. Loss of FA signaling resulted in increased aortic stiffness [17]. Reduced expression of miR-92a with age also was shown to associate with increased aortic stiffness [84]. A few other studies also reported age dependent increase in aortic stiffening caused by epigenetic alterations. However, an increase in ECM stiffness [85] was shown as the underlying molecular mechanism responsible for the observed effect. Further studies are required to understand how epigenetic alterations affect the VSMC cytoskeleton with aging and their impact on aortic stiffness.

## Changes in VSMC function with aging and implications for potential therapeutic target development

**Figure 2.**
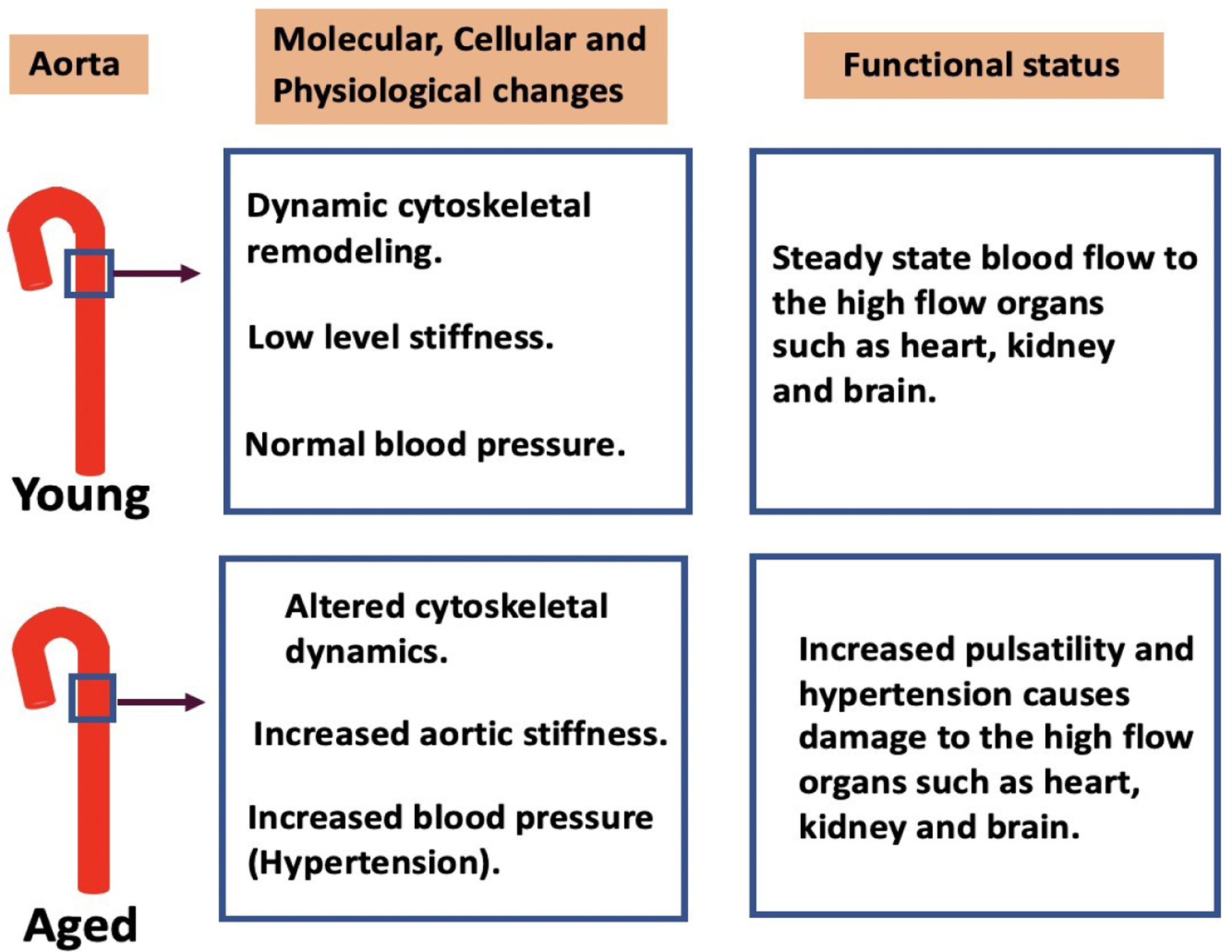
Cytoskeletal dynamics in the aorta with ageing and functional consequences. In young aortas dynamic cytoskeletal remodeling maintains low levels of stiffness and normal blood pressure which allow normal blood flow to the high flow organs such as heart, kidney, and brain. With advancing age, a decrease in cytoskeletal dynamics increases the stiffness of the aortic wall. This causes an increased pulsatility of the blood flow leading to damage of the end organs. Therapeutic interventions are required to prevent age-related adverse cardiovascular effects associated with vascular stiffness. Peptides that reduce stiffness by blocking cytoskeletal interactions or other novel approaches are of interest as future potential therapeutics

In the young adult mouse model, interactions between components of the cytoskeleton are dynamic, transient and reversible and do not lead to lasting changes in stiffness (Figure 2). But, as described above, our group has found that in aged animals, some cytoskeletal components become attached in a sustained manner, increasing the stiffness of the tissue. As a possible first step in a therapeutic approach, synthesized peptide decoy inhibitors of cytoskeletal stiffness have been produced [18] to try to reverse aging-induced increases in vascular stiffness. The peptides have been made as mimics of part of the protein sequence of cytoskeletal proteins, such as talin or vinculin, but lacking a binding site to their downstream effector. In this way they compete with endogenous molecules and prevent those molecules from bridging parts of the cytoskeleton. This results in a significant decrease in aortic stiffness, at least as tested *ex vivo*.

It is important to point out that these decoy peptides were designed to be targeted to the aorta but, in theory, it should also be possible to design a variant of these peptides to target molecular interactions in the peripheral vasculature or the resistance vessels and to decrease blood pressure rather than aortic stiffness. The vasoactive peptide approach is effective in decreasing vascular contractility and hence could be useful to tackle hypertension, but a means of successfully targeting the peptides to specific resistance vessel beds would need to be developed. Nicholson et al. [18] demonstrated that the peptides can be loaded onto microbubbles for tissue delivery, and subsequently burst by ultrasound to load the peptides into VSMCs. However, considerable work is needed to determine if ultrasound targeting of these peptide-loaded microbubbles can be used effectively *in vivo*, particularly in the human.

## Conclusions and future approaches

### Value of cytoskeletal targets for future drug development

Thus far few, if any, cytoskeletal targets have been identified for the development of potential therapeutics to treat or prevent hypertension or aortic stiffness. However, the studies cited above provide a limited proof of concept that such targets may be useful as prototype therapeutic approaches for the treatment of, not only hypertension, but also aortic stiffness associated with aging.

### Future approaches

Recently, basic science investigators interested in molecular mechanisms of arterial stiffness have asked the question of whether sex-specific differences exist. Indeed, many differences between the sexes have been reported [86, 87], at least in animal models, and this line of investigation should be expanded. However, as mentioned above, because of species-specific differences, any broad conclusions between human and animal models on sex-related differences need to be made with great caution.

As most of the published aortic stiffness research work has used cells and tissues obtained from non-human origin, it may not directly lead to therapeutic interventions to treat increased aortic stiffness in humans. Cells and tissues derived from human subjects may provide better insights into understanding the age dependent increase in aortic stiffness but are difficult to obtain. Patient-derived induced pluripotent stem cells (iPSCs) may provide a way to study vascular diseases as these cells can carry pathological features similar to those in the human tissues [88]. Multiple different induction media and protocols have been used to derive VSMCs from patient iPSCs [89]. This has been a useful approach to model for certain vascular pathologies such as Hutchison Gilford Progeria Syndrome (HGPS) [90, 91], atherosclerosis [92], aortic aneurysm [93], supravalvular aortic stenosis [94], and hypertension [95]. Though there are challenges associated with generation of lineage-specific and mature VSMCs from iPSCs that match the *in vivo* counterparts [88, 89], iPSC-VSMCs could help understand the molecular mechanisms associated with aortic stiffening and to screen drugs for potential therapeutic interventions.

## Abbreviations

ECM: extracellular matrix
ERK: extracellular signal-regulated kinase
FA: focal adhesion
FAK: focal adhesion kinase
iPSCs: induced pluripotent stem cells
MEK: mitogen-activated protein kinase kinase
miRs: microRNAs
MLCK: myosin light chain kinase
Raf: rapidly accelerated fibrosarcoma
TET: Ten-eleven translocation
VSMC: vascular smooth muscle cell
